# Investigating the Potential Influence of Cause of Death and Cocaine Levels on the Differential Expression of Genes Associated with Cocaine Abuse

**DOI:** 10.1371/journal.pone.0117580

**Published:** 2015-02-06

**Authors:** Michael J. Bannon, Candace L. Savonen, Zachary J. Hartley, Magen M. Johnson, Carl J. Schmidt

**Affiliations:** 1 Department of Pharmacology, Wayne State University School of Medicine, Detroit, Michigan, United States of America; 2 Department of Pathology, University of Michigan Medical School, Ann Arbor, Michigan, United States of America; Harvard Medical School, UNITED STATES

## Abstract

The development of new therapeutic strategies for the treatment of complex brain disorders such as drug addiction is likely to be advanced by a more complete understanding of the underlying molecular pathophysiology. Although the study of postmortem human brain represents a unique resource in this regard, it can be challenging to disentangle the relative contribution of chronic pathological processes versus perimortem events to the observed changes in gene expression. To begin to unravel this issue, we analyzed by quantitative PCR the midbrain expression of numerous candidate genes previously associated with cocaine abuse. Data obtained from chronic cocaine abusers (and matched control subjects) dying of gunshot wounds were compared with a prior study of subjects with deaths directly attributable to cocaine abuse. Most of the genes studied (i.e., tyrosine hydroxylase, dopamine transporter, forkhead box A2, histone variant H3 family 3B, nuclear factor kappa B inhibitor alpha, growth arrest and DNA damage-inducible beta) were found to be differentially expressed in chronic cocaine abusers irrespective of immediate cause of death or perimortem levels of cocaine, suggesting that these may represent core pathophysiological changes arising with chronic drug abuse. On the other hand, chemokine C-C motif ligand 2 and jun proto-oncogene expression were unaffected in cocaine-abusing subjects dying of gunshot wounds, in contrast to the differential expression previously reported in cocaine-related fatalities. The possible influence of cause of death and other factors on the cocaine-responsiveness of these genes is discussed.

## Introduction

Drug addiction is associated with persistent changes in neural gene expression thought to arise through a number of transcriptional and chromatin-related mechanisms [1]. A number of discrete yet interacting neural circuits are demonstrably involved in the addiction process; among these, midbrain dopamine (DA)-synthesizing neurons certainly play a featured role. DA neurons mediate not only acute rewarding effects of drugs of abuse but conditioned responses to cues associated with past drug use [2]. Chronic drug (particularly stimulant) abuse may also elicit deficits in DA neurotransmission that contribute to the adverse states associated with the cessation of drug use, although this process is less well-understood [3]. A more complete understanding of the molecular pathophysiology occurring in drug-exposed DA cells could suggest new therapeutic targets for the treatment of drug abuse. Postmortem human brain represents a unique resource for gaining insights into complex disorders such as drug abuse [4]. In a recent analysis of human postmortem midbrain samples from subjects who had died of cocaine abuse, we identified a molecular profile of differential expression of genes that regulate transcription, chromatin, and DA cell phenotype [5].

One challenge in interpreting human postmortem data pertains to the relative contributions of perimortem events as opposed to chronic pathological processes in eliciting the changes in gene expression observed. As a step toward unraveling this issue, in the present study we analyzed (by quantitative PCR) midbrain samples from a group of chronic cocaine abusers who died as a result of gunshot wounds. We then compared these data with data from a prior study of subjects with deaths directly attributable to cocaine abuse [5], a strategy we have previously used to analyze cocaine-responsive genes in the human nucleus accumbens [6]. In the current experiments, we confirmed that numerous genes previously identified as cocaine-responsive were, in fact, differentially expressed irrespective of immediate cause of death or perimortem levels of cocaine, suggesting that they reflect some core pathophysiological changes arising in the midbrain DA neurons of cocaine abusers. On the other hand, a few genes differentially expressed in our prior study were unaffected in the current group of cocaine-abusing gunshot fatalities. The cocaine-responsiveness of these genes may be influenced by cause of death or other perimortem factors, though further studies are needed to clarify this issue. The ability to distinguish between gene expression changes that are robustly diagnostic for chronic cocaine abuse and those changes that are more variable in nature and/or affected by perimortem factors could impact the future development of both therapeutic approaches for cocaine abuse and forensic assays for the determination of cocaine-related fatalities.

## Materials and Methods

### Ethics statement

This study utilized de-identified cadaver specimens obtained at routine autopsy. The National Institutes of Health (DHHS) has determined that the use of such specimens does not constitute human subjects research and is not subject to regulations pertaining to Human Subjects Research (45 CFR part 46).

### Case selection

Human midbrain specimens were collected during the routine autopsy process and characterized as described in detail [5–10]. Briefly, cause of death was determined by forensic pathologists following medico-legal investigations evaluating the circumstances of death including medical records, police reports, autopsy results, and toxicological data. All cases used in this study died from gunshot wound(s). Case inclusion in the cocaine group (n = 10) was based on a documented history of drug abuse and a toxicology positive for cocaine and cocaine metabolites but negative for other drugs of abuse or CNS medications at time of death (with the exception of low levels of ethanol [0.02–0.04 g/dl] in two cases). Cases included in the control group (n = 8) had no documented history of drug abuse and tested negative for cocaine, cocaine metabolites, and other drugs of abuse and CNS medications (with the exception of 0.04 g/dl ethanol in a single case). Cases were not screened for the presence of nicotine or metabolites. Exclusion criteria included a known history of neurological or psychiatric disorder, evidence of neuropathology (e.g. stroke, encephalitis) or chronic illness (e.g. cirrhosis, cancer), death by suicide, a prolonged survival period between shooting and death, or an estimated postmortem interval >20 hr. To reduce any variance between groups unrelated to drug abuse, the two groups were matched in terms of gender, race, age, and well-established measures of tissue sample quality and perimortem agonal state (i.e. brain pH and RNA integrity number [RIN]; [11,12]).

### Sample processing, RNA extraction, quantitative PCR, and statistical analysis

All methodologies have been previously described in detail [5,9,10]. Briefly, fresh-frozen postmortem samples encompassing the ventral midbrain were cryosectioned, and DA cell-enriched regions finely dissected and pooled. RNA was isolated, quantified, and assessed for integrity. Transcript abundance was quantified for a number of genes previously identified as differentially expressed in cocaine abusers, using published quantitative PCR methods and primer sequences [5]. Statistical analyses (independent t-tests, Pearson’s correlations) were conducted using SPSS or MS Excel software.

## Results and Discussion

**Fig 1:**
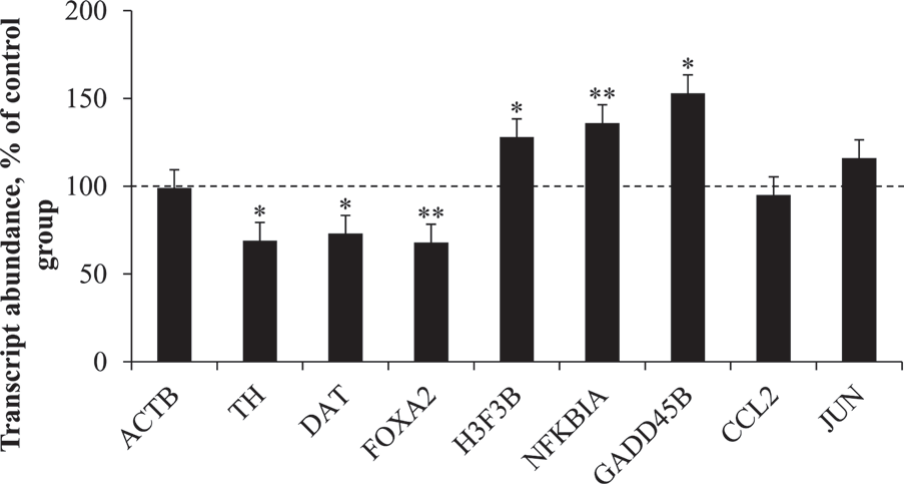
Differential gene expression in the midbrain of human cocaine abusers . Transcript abundances were quantified in specimens from cocaine abusers and control subjects. All subjects died of gunshot wounds (see Table 1 as well as Materials and Methods for case characteristics). Data from cocaine abusers are expressed as a percentage (mean + SEM) of control subjects. * *p*<0.05; ** *p*<0.005 by 1-tailed t-test for independent means. Abbreviations: ACTB, actin; TH, tyrosine hydroxylase; DAT, dopamine transporter; FOXA2, forkhead box A2; H3F3B, histone variant H3, family 3B; NFKBIA, nuclear factor kappa B inhibitor alpha; GADD45B, growth arrest and DNA damage-inducible beta; CCL2, chemokine C-C motif ligand 2; JUN, jun proto-oncogene.

Postmortem specimens of DA cell-enriched ventral midbrain were obtained from chronic cocaine abusers and drug-free controls, all of whom had died as a result of gunshot wound(s). These groups did not differ in terms of either subject characteristics (i.e. gender, age, race) or well-established measures of tissue sample quality and perimortem agonal state (i.e. brain pH and RIN)(for details, see Materials and Methods & Table 1). As expected, midbrain actin (ACTB) gene expression, as determined by quantitative PCR, was indistinguishable between the two groups (Fig. 1).

**Table 1:** Case characteristics.

	Cause of Death	Age	Race/Sex	Cocaine (μg/mL)	Benzoylecgonine (μg/mL)	Brain pH	RIN
Control Cases	GSW	20	BM	0	0	6.9	7.3
	MGSW	25	BM	0	0	6.5	6.7
	MGSW	25	BM	0	0	6.8	6.9
	GSW	30	BM	0	0	6.7	6.2
	GSW	34	WM	0	0	6.8	7.0
	GSW	37	BM	0	0	6.8	7.1
	MGSW	39	BM	0	0	6.4	7.1
	MGSW	45	BM	0	0	6.8	6.7
Mean ± S.E.		32 ± 8		0 ± 0	0 ± 0	6.7 ± 0.05	6.9 ± 0.11
Cocaine Cases	MGSW	25	BM	0.76	1.70	6.6	6.5
	GSW	25	BM	<0.025	1.50	6.5	6.7
	MGSW	30	BM	0.70	2.70	6.8	7.0
	GSW	34	BM	<0.025	0.84	6.5	7.4
	MGSW	34	WM	0.05	1.60	6.7	6.8
	GSW	34	WM	0.04	4.20	6.3	7.2
	MGSW	35	BM	0.17	1.10	6.7	6.8
	GSW	36	BM	0.04	0.53	6.7	6.6
	MGSW	38	BM	0.19	3.50	6.3	6.8
	GSW	40	BM	0.16	1.80	6.5	7.7
Mean ± S.E.		33 ± 5		0.26 ± 0.29	1.7 ± 0.98	6.6 ± 0.05	6.9 ± 0.11

Abbreviations: BM, black male; GSW, gunshot wound; MGSW, multiple gunshot wounds; RIN, RNA integrity number; WM, white male.

From among the small number of genes that we’d previously found were differentially expressed in the midbrains of cocaine-related fatalities [5], we chose for this study a subset of genes both enriched in DA cell expression and representative in terms of the magnitude of change observed and primary biological processes affected. The expression of three genes strongly associated with midbrain DA cell phenotype, namely tyrosine hydroxylase (TH), DA transporter (DAT, aka SLC6A3), and forkhead box A2 (FOXA2), was significantly down-regulated (p<0.05 to p<0.005) in cocaine abusers relative to control cases (Fig. 1), in keeping with our previous report [5]. Likewise, the expression of three genes associated with chromatin-mediated or transcriptional regulation of gene expression, namely histone variant H3 family 3B (H3F3B), nuclear factor kappa B inhibitor alpha (NFKBIA), and growth arrest and DNA damage-inducible beta (GADD45B), was up-regulated (p<0.05 to p<0.005) in cocaine abusers (Fig. 1), as described [5].

**Fig 2:**
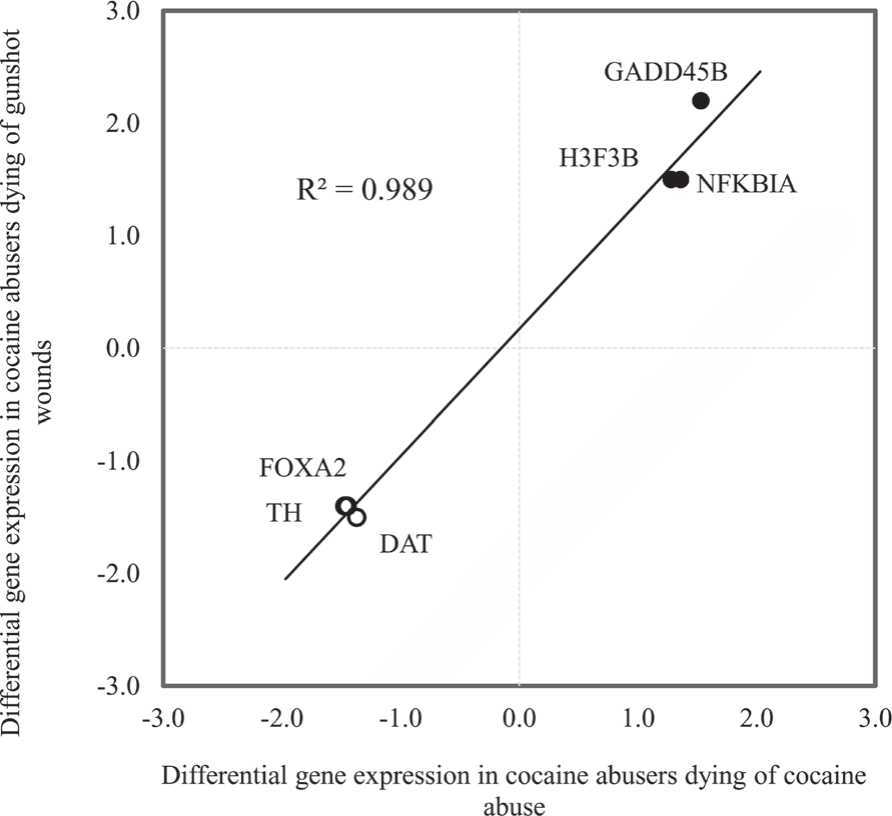
Correlation between the differential gene expression seen in cocaine abusers independent of cause of death. Differentially expressed genes from Fig. 1 are plotted against published microarray data from a different cohort of cocaine abusers [5] as fold-differences. Pearson’s correlation R^2^ = 0.99, p<0.001.

As shown in Fig. 2, the magnitude of differential expression of these six genes correlated significantly (R^2^ = 0.99; p<0.001) with that seen previously in a different cohort of cocaine abusers [5]. Down-regulation of TH, DAT, and FOXA2 gene expression and up-regulation of H3F3B, NFKBIA, and GADD45B gene expression are thus evident in chronic cocaine abusers’ midbrains independent of cause of death (i.e. cocaine abuse versus gunshot wounds). Given cocaine’s short half-life, the presence of unmetabolized cocaine in blood (Table 1) provides evidence of active cocaine use shortly before death. Nevertheless, the levels of cocaine (and its major metabolite benzoylecgonine) did not correlate with the abundance of these transcripts (Table 2), arguing that recent cocaine use is not a major determinant of the differential expression of these genes. Overall, the data are consistent with the interpretation that these changes in midbrain gene expression reflect pathophysiological processes associated with chronic cocaine abuse.

**Table 2:** Absence of correlation between transcript abundances and cocaine or benzoylecgonine levels.

		ACTB	TH	DAT	FOXA2	H3F3B	NFKBIA	GADD45B	CCL2	JUN
Cocaine	R value	-0.15	-0.08	0.04	-0.13	-0.56	-0.51	-0.36	-0.43	-0.50
	p-value (2 tailed)	0.69	0.83	0.91	0.73	0.09	0.13	0.31	0.21	0.14
Benzoylecgonine	R value	0.20	-0.11	-0.07	-0.07	-0.30	-0.58	0.55	0.47	0.58
	p-value (2 tailed)	0.59	0.76	0.84	0.86	0.39	0.08	0.10	0.17	0.08

In this study, neither chemokine C-C motif ligand 2 (CCL2) nor jun proto-oncogene (JUN) gene expression differed as a function of cocaine abuse in gunshot victims (Fig. 1), in contrast to the large differential expression seen previously in subjects dying of cocaine abuse [5]. The reason(s) for this discrepancy are unknown. In terms of demographics, the cocaine cohort in this study were younger on average than those previously described (33±5 versus 50±1 years of age), so an interaction between subject age and the effects of cocaine on CCL2 and JUN cannot be ruled out. In terms of perimortem variables, CCL2 and JUN transcript abundances were unrelated to cocaine levels (Table 2). Likewise, although we’ve previously shown that a prolonged period of survival with medical/law enforcement interventions can affect some CNS gene expression [9], cases with this profile were intentionally excluded from the current study. We also cannot exclude the intriguing possibility that midbrain CCL2 and JUN gene expression are not induced by ongoing processes associated with chronic drug abuse, but rather by some unknown perimortem factor(s) associated with cocaine abuse as the cause of death *per se*. Clearly, further studies with larger, more varied cohorts will be required to determine if the differential expression of CCL2 or JUN might provide biomarkers for cocaine-related fatalities.

A number of limitations associated with the study warrant mention. The sample size employed is modest, largely due to efforts to carefully match the cocaine-abusing and control groups in terms of numerous demographic and sample quality parameters. In addition, this study compares data from a new cohort of cocaine-abusing gunshot victims with previously published data from cocaine-related fatalities; differences between the datasets should therefore be interpreted with a degree of caution. Given these limitations, the strong commonalities in gene expression evident between the two cohorts of cocaine abusers, independent of cause of death, cocaine levels, and subject age, reinforce the conclusion [5] that a molecular profile of chronic cocaine abuse includes differential midbrain expression of genes regulating chromatin, transcription, and DA cell phenotype (Fig. 1). In terms of the genes that we confirmed were up-regulated in cocaine abusers, H3F3B encodes a replication-independent histone variant thought to represent an epigenetic imprint of transcriptionally active chromatin [13], GADD45B encodes a protein that regulates neuroplasticity and neural gene promoter DNA demethylation [14,15], and NFKBIA encodes a modulator of the transcription factor NFKB implicated in drug addiction [16]. Changes in the expression of these genes most likely exert downstream effects on many other neural genes. In terms of the genes down-regulated in cocaine abusers, FOXA2 encodes a critical DA cell-specifying transcription factor [17], TH encodes the rate-limiting enzyme in DA biosynthesis, and DAT encodes the primary regulator of extracellular DA levels and target of cocaine binding [18]. These data confirm that cocaine abuse has a rather broad impact on the expression of the midbrain DA cell phenotype [5] which may, in turn, exacerbate decrements in DA signaling that emerge during the normal human aging process [19, 20]. The identification of numerous genes robustly and reproducibly affected by cocaine abuse independent of immediate cause of death or cocaine levels provides some additional insights into the underlying cellular pathophysiology of drug abuse, which could ultimately contribute to the development of novel therapeutic approaches.
